# Hindlimb suspension in Wistar rats: Sex‐based differences in muscle response

**DOI:** 10.14814/phy2.15042

**Published:** 2021-10-06

**Authors:** Marie Mortreux, Megan E. Rosa‐Caldwell, Ian D. Stiehl, Dong‐Min Sung, Nicholas T. Thomas, Christopher S. Fry, Seward B. Rutkove

**Affiliations:** ^1^ Department of Neurology Harvard Medical School – Beth Israel Deaconess Medical Center Boston Massachusetts USA; ^2^ Department of Physics and Astronomy Dartmouth College Hanover New Hampshire USA; ^3^ Department of Athletic Training and Clinical Nutrition University of Kentucky Lexington Kentucky USA

**Keywords:** ground‐based, microgravity, muscle, sex‐based, spaceflight

## Abstract

Ground‐based animal models have been used extensively to understand the effects of microgravity on various physiological systems. Among them, hindlimb suspension (HLS), developed in 1979 in rats, remains the gold‐standard and allows researchers to study the consequences of total unloading of the hind limbs while inducing a cephalic fluid shift. While this model has already brought valuable insights to space biology, few studies have directly compared functional decrements in the muscles of males and females during HLS. We exposed 28 adult Wistar rats (14 males and 14 females) to 14 days of HLS or normal loading (NL) to better assess how sex impacts disuse‐induced muscle deconditioning. Females better maintained muscle function during HLS than males, as shown by a more moderate reduction in grip strength at 7 days (males: −37.5 ± 3.1%, females: −22.4 ± 6.5%, compared to baseline), that remains stable during the second week of unloading (males: −53.3 ± 5.7%, females: −22.4 ± 5.5%, compared to day 0) while the males exhibit a steady decrease over time (effect of sex × loading *p* = 0.0002, effect of sex × time × loading *p* = 0.0099). This was further supported by analyzing the force production in response to a tetanic stimulus. Further functional analyses using force production were also shown to correspond to sex differences in relative loss of muscle mass and CSA. Moreover, our functional data were supported by histomorphometric analyzes, and we highlighted differences in relative muscle loss and CSA. Specifically, female rats seem to experience a lesser muscle deconditioning during disuse than males thus emphasizing the need for more studies that will assess male and female animals concomitantly to develop tailored, effective countermeasures for all astronauts.

## Graphical abstract

Using the well‐established hindlimb suspension model in rats, we find females appear to have attenuated muscle decrements compared to males after 14 days of disuse. Specifically, females have attenuated decrements to muscle size, cross‐sectional area, and force productions compared to males. These results provide more nuances on the possible interactions between sexes during muscle atrophies and emphasize the importance of addressing sex‐based differences in research investigations, both on the ground and in space.

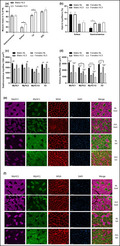

## 1 INTRODUCTION

Long duration spaceflight has been well established to produce substantial muscle loss and reduced functional capability. For example, data in astronauts from the international space station (ISS) have shown ~3% loss in muscle mass for every month in microgravity (LeBlanc, Lin, et al., 2000; LeBlanc, Schneider, et al., 2000), with the greatest loss observed in triceps surae and quadriceps muscles (Fitts et al., 2000), that are critical for normal ambulation. Upon return to Earth, this loss of lean mass is associated with reduced health outcomes such as increased risk for injury and chronic low‐back pain (Pool‐Goudzwaard et al., 2015; Stein, 2013; Wing et al., 1991). Recently, interventions, such as prescribed exercise using specialized equipment, have been implemented to mitigate microgravity‐induced muscle loss (Petersen et al., 2016; Sibonga et al., 2019; Trappe et al., 2009; Winnard et al., 2017). While these new interventions attenuate musculoskeletal loss (Sibonga et al., 2019), they do not fully maintain muscle mass (Tanaka et al., 2017; Trappe et al., 2009). More so, future missions will undoubtedly entail exposure to microgravity exceeding 1 month and may afford more limited exercise countermeasure opportunities.

Despite much research into the impact of microgravity on muscle health (Fitts et al., 2001), comparatively little is known about how biological sex impacts interacts with the development of muscle atrophy in this context (Mark, 2007; Ploutz‐Snyder et al., 2014). Recent literature has speculated that physiological differences may affect progression of muscle atrophy (Rosa‐Caldwell & Greene, 2019; Zhong & Zimmers, 2020). For example, female mice are less susceptible to cancer cachexia than male (Hetzler et al., 1852; Lim et al., 2020). Specific to microgravity, recent studies have shown that female mice have more dramatic alterations to moderators of muscle size, including lower protein synthesis rates and greater induction of protein degradative pathways within the early stages of disuse compared to males (Rosa‐Caldwell et al., 2021). In rat studies, 2 weeks of unloading has been shown to result in greater muscle atrophy in males than females (Deschenes & Leathrum, 2016). Moreover, males exhibit a lesser decrement in muscle strength initially but ultimately tend to be more prone to fatigue than females. Conversely, protocols using 30‐day hindlimb unloading in rats failed to detect sex differences in muscle deconditioning and atrophy (Il’ina‐Kakueva, 2002). Similar studies have been conducted in humans exposed to 1 week of unloading and revealed that women had greater declines in muscle performance and neuromuscular functions despite suffering a similar degree of muscle atrophy (Deschenes et al., 2009, 2012). Other investigations using arm suspension showed that unloading appeared to more dramatically affect maximal isometric force production in females; yet muscular endurance in females was not affected after disuse (Miles et al., 2005).

Finally, several studies have assessed muscle stem cell (i.e., satellite cell) activity during disuse given their contribution to muscle plasticity. While studies have shown perturbations in both activity and abundance (McKenna & Fry, 2017), additional work has highlighted the existence of strong sex‐based differences. Studies in mice have shown that throughout their life, males possess a greater abundance of satellite cells compared to females (Neal et al., 2012), and research conducted in other animal models demonstrates the effect of sex and sexual hormones on the proliferation and differentiation of satellite cells (Lee et al., 2011; Song et al., 2013). Taken together, there is plausible evidence that males and females may have different developmental patterns for the development of disuse atrophy and response to therapy or prevention. Despite these differences, there has been a dearth of head‐to‐head comparisons of muscle functional differences in males and females in the development of atrophy. In this article, we sought to specifically address that question using the hindlimb suspension (HLS) model in adult rats.

## 2 MATERIALS AND METHODS

### 2.1 Animals

All experimental protocols were approved by the Beth Israel Deaconess Medical Center Institutional Animal Care and Use Committee under the protocol #025‐2019. Twenty‐eight Wistar rats (14 males and 14 females, 14‐weeks old at baseline) were obtained (Charles River Laboratories) and housed separately in a temperature‐controlled room (22 ± 2℃) with a 12:12h light‐dark cycle starting at 7:00 AM. Water and chow were provided ad libitum and food intake was recorded daily. Rats were acclimated to their custom‐cage for 48 h before experiments started. Groups were composed of 6–8 animals and body weights were matched within each sex at the start of the experiment (416 ± 4 g for the males, 261 ± 4 g for the females). For all experiments requiring anesthesia, inhaled isoflurane (1.5–3.5%) + oxygen was used, and the animal was placed in a prone position with the left hindlimb taped at a 45° angle and the fur was removed using clippers.

### 2.2 Hindlimb suspension (HLS)

Animals were placed in either hindlimb suspension (HLS) using a pelvic harness as previously described (Mortreux et al., 2019b, 2020) or at normal loading (NL) in identical cages for 14 days. Briefly, for HLS, animals were placed in a fitted pelvic harness secured to the top of the cage by a stainless‐steel chain. The chain was then adjusted until the animal's hind limbs could not reach the floor. Animals were singly housed in identical cages described elsewhere (Mortreux et al., 2018), and cotton squares were provided as enrichment.

### 2.3 Voluntary grip force

Using a 50 Newton capacity digital grip force meter (Chatillon), rats’ front paws were placed on the grip bar and the animal pulled backwards until it released its grip; the peak force applied was recorded. Rats underwent three trials with a short latency period (~30 s) and both the average and the peak force were recorded. For rear paw grip force, the animal was held near the shoulders and the animal's rear paws placed on the grip force bar. After the researcher confirmed both paws were gripping the force bar, the animal was pulled from the bar until it released its grip. Grip force measurements were performed at baseline (pre‐suspension) and weekly thereafter.

### 2.4 Calf circumference

Calf circumference was obtained using a suture thread at the tibial mid‐shaft in animals under anesthesia. Three separate measurements were obtained and the average was recorded.

### 2.5 Maximal tetanic force production

Weekly, rats were anesthetized and placed in a supine position on a force plate (Dual Mode Muscle Lever System; Aurora Scientific), with their left foot taped securely and their knee stabilized. Care was taken such that the animal was positioned to ensure maximal tetanic force, including alignment of the patella and the ankle joint, 0° of knee flexion, and 0° of dorsiflexion (i.e., 90° angle between the foot and the tibia). To ensure proper placement of the monopolar electrodes (28G; Natus Medical Inc.), a 10 Hz twitch was used to determine appropriate position. Thereafter, a tetanic, supramaximal stimulation was delivered to the tibial nerve (for plantar flexion) or to the peronal nerve (for dorsal flexion), at a frequency of 200 Hz, for 200 ms. The maximum torque was recorded and data were converted to absolute values.

### 2.6 Sample collection and tissue harvest

Food intake was assessed daily each morning. Weekly, animals’ body weight was recorded. Blood samples were collected between 8:30 and 8:45 a.m. using a tail nick in awake animals. Samples were then centrifuged and plasma was collected, logged, and stored at −20℃.

At the end of the experiment, rats were euthanized by CO_2_ inhalation according to IACUC guidelines. Cardiac puncture was performed and organs were excised and weighed using a precision analytic scale (Fisher Scientific). Blood from the cardiac puncture was allowed to clot at room temperature and then centrifuged for 10 min at 18,000 *g*, and serum was collected, aliquoted, and stored at −20℃. Left‐side muscles were then placed in 10% neutral buffered formalin for 48 h at 4℃ (including gastrocnemius and soleus), and transferred to 1× PBS for further processing. The right‐side soleus muscles were excised and immediately frozen in liquid nitrogen and stored at −80℃.

### 2.7 Muscle histology

The left gastrocnemius and soleus muscles were embedded in paraffin and immunohistochemical analysis was performed on full cross‐sections from the muscle belly. Labelling was performed using primary antibodies directed against slow‐skeletal myosin heavy chain (ab11083; Abcam), fast‐skeletal myosin heavy chain (ab91506; Abcam) and wheat germ agglutinin (W6748; Thermofisher Scientific). Images were then acquired using an epifluorescence microscope at 20× magnification (Zeiss Axio Imager M1), and analyzed with FIJI (ImageJ, NIH) to determine myofiber cross‐section area (CSA) with the help of the muscle morphometry plug‐in (by Anthony Sinadinos using Eclipse IDE); and the experimenter was blinded for treatment during picture acquisition and CSA measurement. For all measurements, the entire cross‐section of both the gastrocnemius and soleus, and consisted of ~200 myofibers per animals. The average of all myofibers within each animal was utilized for inferential statistics.

### 2.8 Pax7 immunohistochemistry and myonuclear density

For Pax7/wheat germ agglutinin/DAPI staining (visualization of satellite cells and myonuclear density), immunohistochemical procedures were adapted from our prior methods (D’Lugos et al., 2019; Finnerty et al., 2017). Briefly, slides were deparaffinized and rehydrated followed by epitope retrieval using sodium citrate (10 mM, pH 6.5) at 92℃ for 20 min.

Endogenous peroxidases were blocked with 3% hydrogen peroxide in PBS, and then slides were incubated overnight at 4℃ in anti‐wheat germ agglutinin conjugated to AF488 tyramide (1:50) (no. W11261; Invitrogen) and anti‐Pax7 antibody (1:100) (Developmental Studies Hybridoma Bank). The next day, slides were incubated for 70 min at room temperature in goat anti‐mouse biotinylated secondary antibody (no. 115‐065‐205; Jackson ImmunoResearch), and then reacted with streptavidin‐horseradish peroxidase (HRP) and AF555 tyramide (1:250) included with the tyramide signal amplification (TSA) kit (#B40933; Invitrogen). TSA‐AF555 was used to visualize Pax7 antibody‐binding. Slides were co‐stained with 4’,6‐diamidino‐2‐phenylindole (DAPI, no. D35471; Invitrogen) before being mounted with Vectashield fluorescent mounting media (Vector Laboratories). Images were captured at 100× total magnification at room temperature with a Zeiss upright microscope (AxioImager M1; Zeiss). Satellite cell abundance was assessed as previously described (D’Lugos et al., 2019; Finnerty et al., 2017). Briefly, Pax7^+^/DAPI^+^ loci residing within the wheat germ agglutinin border were counted as satellite cells by a single assessor blinded to group and sex. Satellite cell abundance was normalized to fiber number for each specimen. Myonuclear density (DAPI + nuclei residing within the wheat germ agglutinin border) was quantified on cross‐sections and normalized to fiber number using MyoVision automated analysis software (Wen et al., 2018).

### 2.9 Immuno‐assays

Plasma from baseline, day 7 and day 14 as well as serum obtained from the cardiac puncture were used to perform ELISA assays to detect estradiol (ab108667; Abcam), creatine‐kinase M (CKM, ab187396; Abcam), and testosterone (mouse rat testosterone, Alpco).

### 2.10 RTqPCR

RNA isolation, cDNA synthesis, and gene analysis were performed as previously described (Greene et al., 2015; Washington et al.,) with minor modifications. Approximately 20 mg of soleus was homogenized in 750 µL of TriZol reagent (Life Technologies, Cat# 15596026) using a commercial homogenizer. Afterwards, 200 µL of chloroform was added to samples and samples were vigorously shaken to incorporate chloroform. Samples were then centrifuged at 20,000 *g* for 25 min at 4℃. The clear supernatant (containing RNA) was carefully aliquoted and the remaining precipitate was discarded. An equal volume of DECP‐treated 70% ethanol was added to aliquoted supernatant. RNA was then isolated from the samples using a commercial kit (Ambion Purelink RNA mini kit, Cat# 12183020; Life Technologies). RNA concentrations and quality were determined using a Nanodrop 1000 Spectrophotometer (Thermo Fisher Scientific). Acceptable RNA quality was determined as a 260/280 ratio between 2.0 and 2.2 per prior reports (Tang et al., 2019). One microgram of RNA was then reverse transcribed into cDNA using commercial reagents (Superscript Vilo, Cat#11755500; Life Technologies). cDNA was then diluted 1:100 for RTqPCR analysis. All samples were analyzed in a 25 µL reaction with Taqman or SYBR master mixes and probes or primers as appropriate. Samples were incubated at 95℃ for 4 min, followed by 40 cycles of denaturation, annealing, and extension at 95, 55, and 72℃, respectively. All experiments were completed with Applied Biosystems 7500 Real‐Time PCR System. Raw Ct values for target genes were analyzed against the housekeeping gene 18S (clone number 4310893E; Thermofisher Scientific) using the 2^−ΔΔ^Ct method as per previous reports (Greene et al., 2015). 18S was ran first to ensure all values did not significantly differ between either males or females (*p* = 0.161) or between HLS and NL animals (*p* = 0.833). Further control was performed to ensure that there were no differences between HLS and NL in males (*p* = 0.422) and females (*p* = 0.748). Primer sequences for target genes are as follows:

*MurF1*F: GGGAACGACCGAGTTCAGACTATC, R: GGCGTCAAACTTGTGGCTCA, *Atrogin* F: AGTGAAGACCGGCTACTGTGGAA, R: TTGCAAAGCTGCAGGGTGAC, *Pax7* F: GATTAGCCGAGTGCTCAGAATCAAG, R: GTCGGGTTCTGATTCCACGTC, *MyoG* F: AACTACCTTCCTGTCCACCTTCA, R: GTCCCCAGTCCCTTTTCTTCCA, *MyoD* F: GAC CCA GAA CTG GGA CAT GGA. R: TGA GTC GAA ACA CGG ATC ATC ATA G, *Ubr5* F: GCACCAATCCTGACGACTCT R: AATGTGTTCTGCTCCGGTCC, *Sirt1* F: GTTGTGTGCCTTCGTTTTGGA R: AGGCCGGTTTGGCTTATACA, *Myf5* F: GGAATGCCATCCGCTACAT, R: GTTACATTCAGGCATGCCGT.

### 2.11 Statistical analyses

All data were analyzed using GraphPad Prism 9.0.2 (GraphPad Software). Longitudinal data were analyzed using three‐way repeated measures ANOVA (factors: time, sex, and loading) followed by Tukey's post hoc test and terminal (e.g., cross‐sectional) analysis were analyzed using two‐way ANOVA (factors: sex and loading) followed by Tukey's post hoc test, and considered significant when *p* < 0.05.

## 3 RESULTS

### 3.1 HLS‐induced greater loss in body weight and grip force in males than females

**FIGURE 1:**
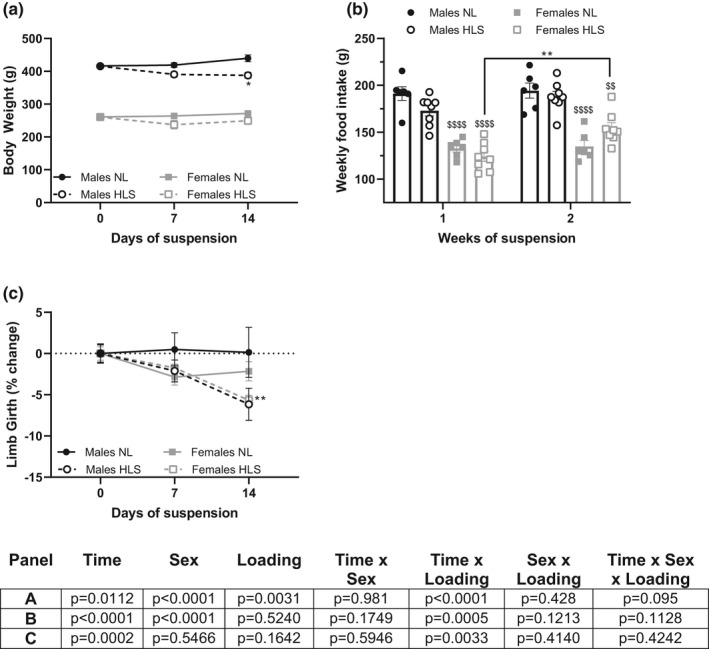
Weekly assessments. Weekly evolution of animals’ body weight (a), food intake (b), and change in limb girth (c), in males (black) and females (gray) exposed to NL (full lines and circles) or HLS (dotted lines and open circles) for 14 days. Results are displayed as mean ± SEM with *n* = 6–8 animals per group. Details of the three‐way ANOVAs can be found under each panel. Tukey's post hoc tests are shown on each panel as *, **: *p* < 0.05 and *p* < 0.01 vs. day 0; $, $$, $$$$: *p* < 0.05, *p* < 0.01, and *p* < 0.0001 between males and females; #, ##: *p* < 0.05 and *p* < 0.01 vs. NL animals. NL: normal loading, HLS: hindlimb suspension

NL animals’ weight increased over the 14‐day period, while HLS rats’ weight decreased (effect of loading: *p* = 0.0031, effect of time × loading: *p* < 0.0001, Figure 1a). At 14 days, HLS males had a significantly lower body weight than at baseline (*p* < 0.05) while females did not. However, weekly food intake remained largely unchanged in our animals (Figure 1b). Males ate significantly more than females but no differences were observed between the food intake of HLS and NL groups. In females, we did not observe significant differences based on loading, however HLS females displayed greater food intake during the second week of unloading than during the first one (*p* < 0.01). Limb girth, a noninvasive indicator of muscle size, remained stable for NL animals (both males and females), but steadily decreased in animals exposed to HLS, with no impact of sex (effect of time × loading *p* = 0.0033, effect of sex × loading *p* = 0.4140, effect of time × sex × loading *p* = 0.4242, Figure 1c).

**FIGURE 2:**
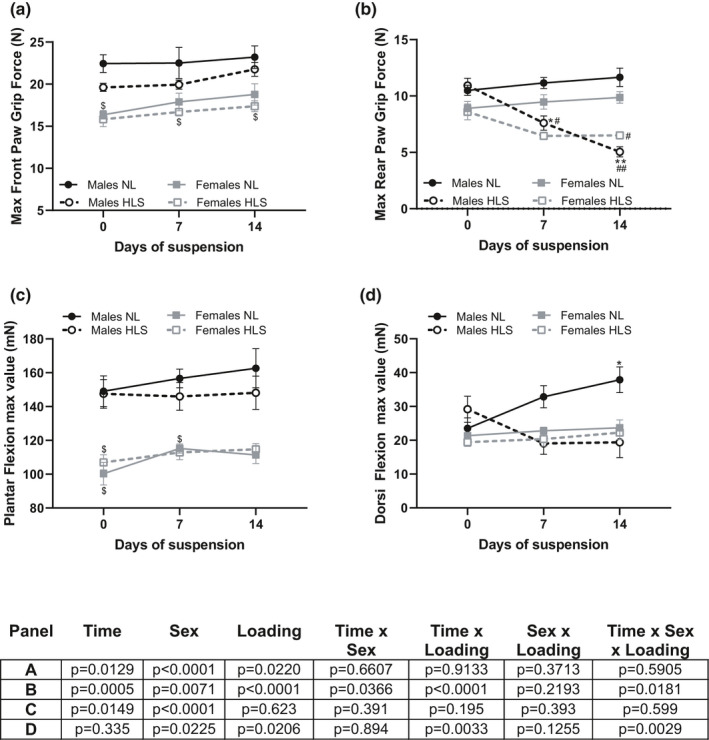
Muscle strength weekly assessment. Maximum front (a) and rear (b) paw grip strength; maximal value recorded during the tetanic stimulation of the tibial nerve to elicit a plantar flexion (c), and during the tetanic stimulation of the peroneal nerve to elicit a dorsiflexion (d); in males (black lines) and females (gray lines) exposed to NL (full lines) or HLS (dotted lines) for 14 days. Results are displayed as mean ± SEM with *n* = 5–8 animals per group. Details of the three‐way Mixed Effect Models and three‐way ANOVAs can be found under each panel. Tukey's post hoc tests are shown on each panel as *, **: *p* < 0.05, *p* < 0.01 vs. day 0; $: *p* < 0.05 between males and females; #, ##: *p* < 0.05, *p* < 0.01 vs. NL animals. NL: normal loading, HLS: hindlimb suspension

Maximal front paw grip force was greater in males than females (effect of sex *p* < 0.0001) but was not significantly impacted by unloading duration (effect of time × loading *p* = 0.9133, effect of time × sex × loading *p* = 0.5905, Figure 2a). On the other hand, maximal hindlimb grip force was impacted by sex, mechanical unloading and the suspension duration (*p* = 0.0071, *p* < 0.0001 and *p* = 0.0005, respectively (Figure 2b). HLS males displayed a linear decline in grip force throughout the experiment, while HLS females did not. At 14 days, both HLS groups displayed values significantly lower than their NL counterparts (effect of time × sex × loading *p* = 0.0181).

### 3.2 HLS resulted in alterations to force generation, which appeared to affect males more than females

During plantar flexion males had greater peak force values than females at baseline (Figure 2c, *p* < 0.05) and these remained higher for the entire protocol duration (effect of sex *p* < 0.0001). Additionally, an effect of time was observed (*p* = 0.0149), whereas loading level did not impact force generation (*p* = 0.623). During dorsiflexion (Figure 2d), males and females did not display differences at baseline. In females, both the NL and HLS group had similar evolution over the 14‐day experiment; however, in males, NL animals produced a significantly greater peak force after 14 days (*p* < 0.05) while the HLS animals did not. Overall, we observed a main effect due to loading (*p* = 0.0206) and identified significant 2 and 3‐way interactions (effect of time × loading *p* = 0.0033, effect of time × sex × loading *p* = 0.0029).

### 3.3 Males experience greater muscle atrophy in response to disuse than females, however, both sexes experience similar reduction in myofiber cross‐sectional area (CSA)

**FIGURE 3:**
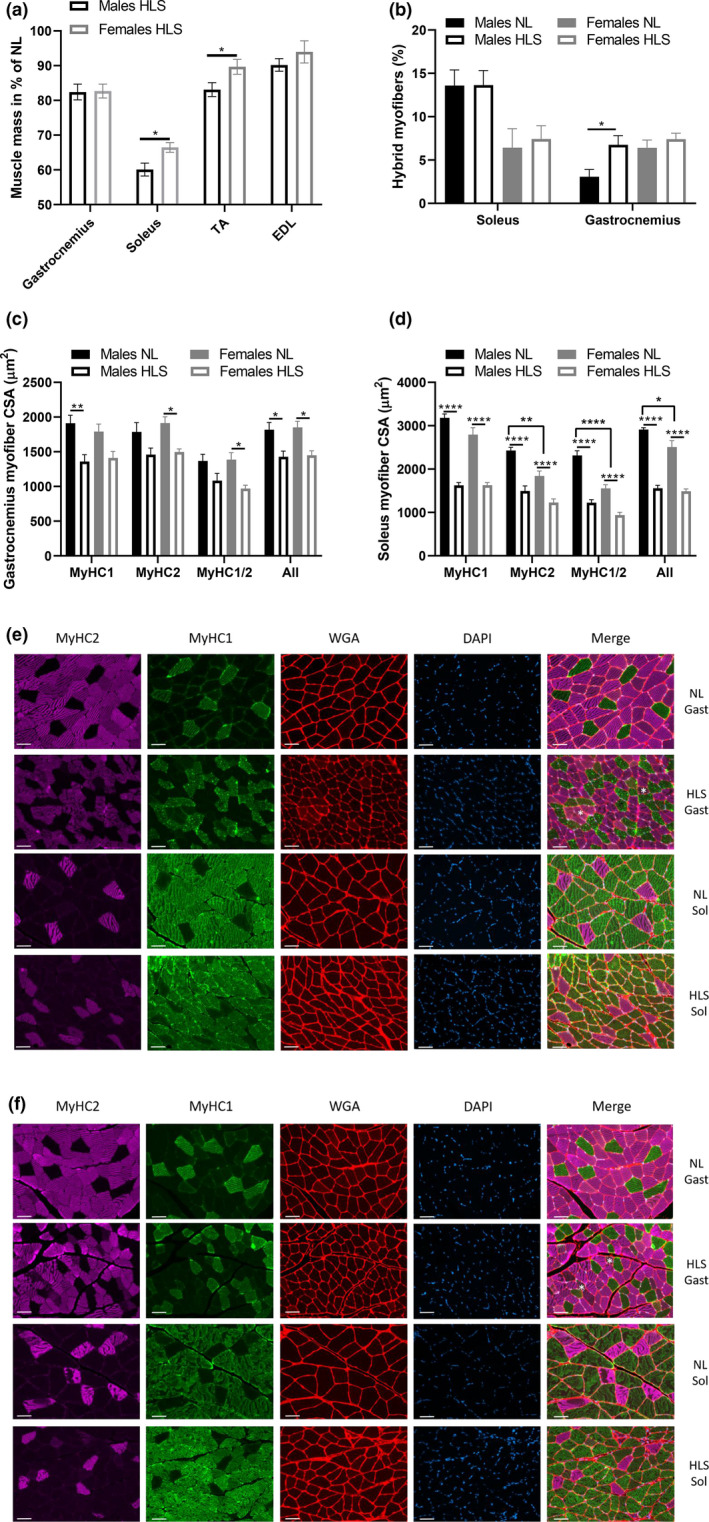
Triceps surae histomorphometry. Hindlimb muscle mass in HLS animals males (black bars) and females (gray bars) compared to NL controls (a), percentage of hybrid myofibers in the soleus and gastrocnemius muscles (b), myofiber CSA per fiber type in the gastrocnemius (c) and in the soleus (d) are displayed. Representative images are displayed for both the males (e) and females (f). Images were acquired at 20x, with the scale bar representing 50 µm. Results are displayed as mean ± SEM with *n* = 5–8 animals per group; and were analyzed using two‐way ANOVA (factors: sex and loading). *, **, ***, ****: *p* < 0.05, *p* < 0.01, *p* < 0.001, and *p* < 0.0001. NL: normal loading, HLS: hindlimb suspension, CSA: cross‐sectional area

After 14 days of hindlimb unloading, HLS animals displayed greater muscle atrophy compared to the NL controls (Table 1 and Figure 3a). In the posterior compartment, gastrocnemius mass was 17.6 ± 2.3% and 17.3 ± 2.0% lower in males and females, respectively, with no difference between sexes. Soleus mass was further reduced in males compared to females (39.9 ± 1.8% vs. 33.5 ± 1.4%, *p* = 0.016). In the anterior compartment, extensor digitorum longus (EDL) mass was 9.8 ± 1.8% and 6.0 ± 3.2% lower in HLS compared to NL in males and females, respectively, with no influence of sex. However, the tibialis anterior (TA) mass was more greatly affected in males than in females (16.9 ± 2.0% vs. 10.3 ± 2.1%, *p* = 0.042).

**TABLE 1:** Hindlimb muscles wet mass

	Males		Females		Two‐way ANOVA		
	NL	HLS	NL	HLS	Loading. *p*	Sex, *p*	Loading × sex, *p*
Gastrocnemius	2.619 ± 0.097	2.158 ± 0.059^c^	1.785 ± 0.036^e^	1.476 ± 0.036 cd	<0.0001	<0.0001	0.215
Soleus	0.223 ± 0.013	0.134 ± 0.004^c^	0.148 ± 0.005^e^	0.098 ± 0.002^ae^	<0.0001	<0.0001	0.006
TA	0.873 ± 0.037	0.725 ± 0.017^b^	0.570 ± 0.016^e^	0.511 ± 0.012^e^	<0.0001	<0.0001	0.048
EDL	0.193 ± 0.009	0.174 ± 0.003	0.128 ± 0.002^e^	0.121 ± 0.004^e^	<0.05	<0.0001	0.275

Results are displayed as mean±SEM and were analyzed using two‐way ANOVA (factors Sex and Loading). *N* = 6–8 per group. Post hoc tests are displayed as a, b, c: *p* < 0.01, *p* < 0.001, and *p* < 0.0001 vs. NL sex‐matched group, d, e: *p* < 0.01 and *p* < 0.0001 vs. loading‐matched males. NL: normal loading, HLS: hindlimb suspension, TA: tibialis anterior, EDL: extensor digitorum longus.

In the gastrocnemius muscle, HLS led to an overall reduction in myofiber CSA (effect of loading *p* < 0.0001), with no impact of sex. However, while HLS males displayed a significant reduction in type 1 (MyHC1) CSA (Figure 3c), females experienced a significant reduction in type 2 (MyHC2) and hybrid myofiber (MyHC1/2) CSA. Moreover, the detection of hybrid myofibers through immunohistochemical staining (Figure 3e), highlighted a significant increase in response to HLS in males (*p* < 0.05) but not females (Figure 3b). In the soleus muscle, overall myofiber CSA was larger in NL males than females (Figure 3d). Unloading resulted in a similar myofiber atrophy in both sexes, regardless of the myofiber type (representative images are available in Figure 3f). Finally, females had less hybrid myofibers than their male counterparts (Figure 3b, *p* = 0.0011).

**FIGURE 4:**
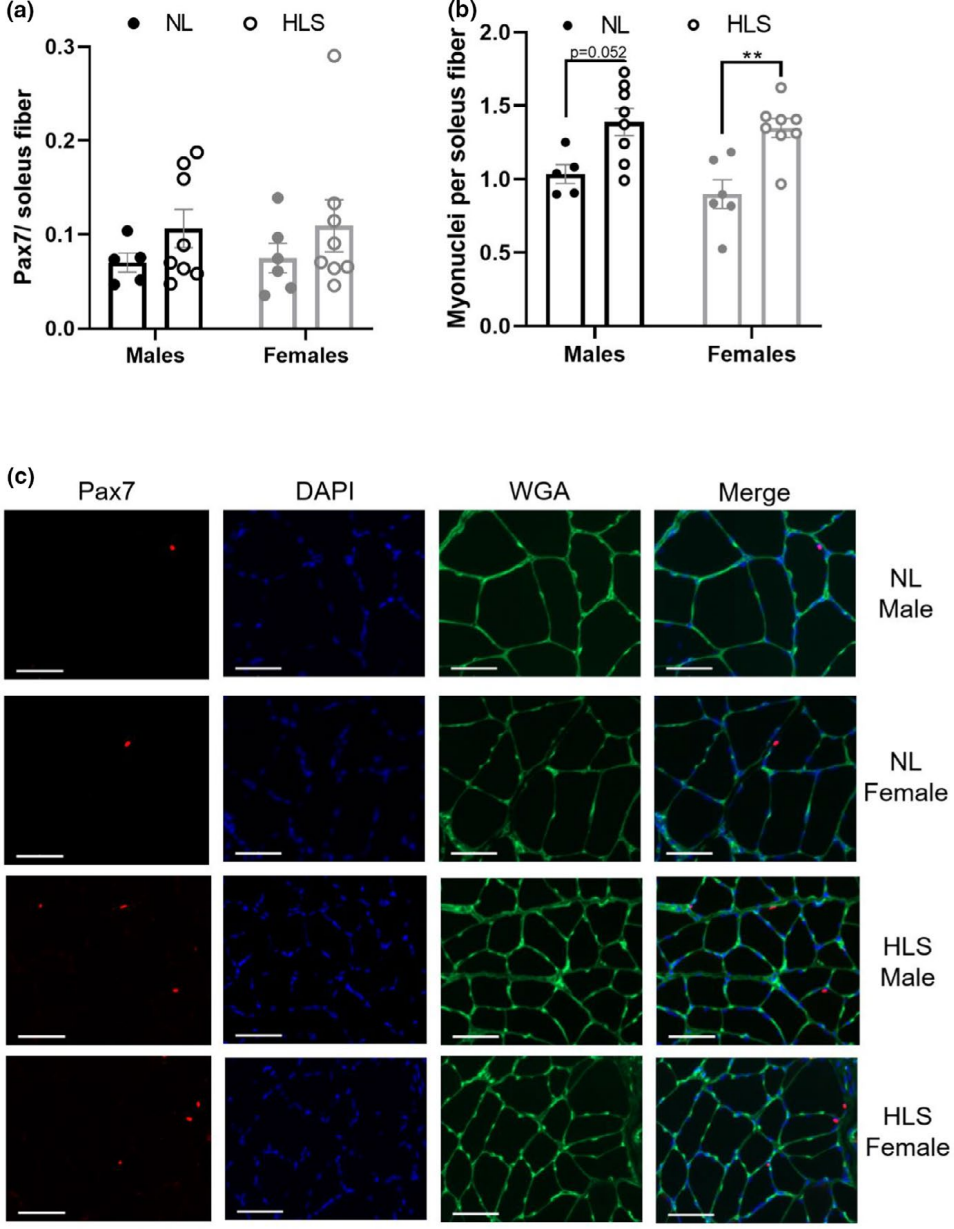
Satellite cells abundance and myonuclear content of the soleus muscle. Immunostaining was performed on the soleus muscle and the Pax7+ cells/fiber (a) and myonuclei density (b) were determined. Representative images are shown (c) and were obtained at 20× with the scale bar representing 50 µm. Results are displayed as mean ± SEM with *n* = 5–8 animals per group; **: *p* < 0.01. NL: normal loading, HLS: hindlimb suspension, WGA: wheat germ agglutinin

Satellite cells were analyzed using Pax7 immunohistochemistry in the soleus muscle and normalized by the number of myofibers (Figure 4). While there was no overall mean difference, we observed a greater (~30%) number of Pax7^+^ cells in animals exposed to HLS (Figure 4a), that did not reach our statistical threshold (*p* = 0.1316). In parallel, myonuclei count was ~45% greater in response to unloading (*p* < 0.0001, Figure 4b). Interestingly, the increased myonuclear density in response to HLS was greater in females (+50.1%, *p* = 0.005) than in males (+34.2%, *p* = 0.052). Representative images for all groups are displayed in Figure 4c.

Plasma testosterone decreased steadily over time in male rats exposed to HLS and was 62% lower at 14 days compared to baseline (*p* = 0.036, Table 2), as compared to NL animal male animals which did not change throughout 14 days of NL. In females, plasma estradiol did not differ in response to loading but we observed an effect of time (*p* = 0.022). Indeed, plasma estradiol concentration decreased during the 2nd week of our experiment to reach 5.6 ± 0.7 and 6.8 ± 0.8 pg/mL in the NL and HLS rats, respectively. In males, HLS led to a nonsignificant increase in CKM concentration (770.25 ± 48.21 vs. 874.22 ± 103.11 in NL and HLS, respectively), while no differences were observed between the females NL and HLS groups.

**TABLE 2:** Serological parameters

Days of suspension		0		7		14	
Parameter	Sex	NL	HLS	NL	HLS	NL	HLS
Testosterone (ng/mL)	Males	2.83 ± 0.49	3.51 ± 0.85	4.92 ± 1.45	2.02 ± 0.58	1.97 ± 0.40	1.33 ± 0.31^#^
	Females	—	—	—	—	—	—
Estradiol (pg/mL)	Males	—	—	—	—	—	—
	Females	13.45 ± 4.72	20.89 ± 7.18	9.77 ± 2.06	11.39 ± 2.25	5.60 ± 0.71	6.77 ± 0.84
Creatine‐Kinase M (ng/mL)	Males	—	—	—	—	770.25 ± 48.21	874.22 ± 103.11
	Females	—	—	—	—	775.00 ± 30.83	772.05 ± 18.73

Results are displayed as mean ± SEM, *n* = 6–8 per group. Testosterone and estradiol were analyzed with two‐way RM ANOVA (factors of time and loading) followed by Tukey's post hoc test. Creatine‐Kinase M (CKM) was analyzed with two‐way ANOVA (factor of sex and loading) followed by Tukey's post hoc test. Testosterone: time × loading *p* = 0.0541, time *p* = 0.0516, loading *p* = 0.1902, #: *p* < 0.05 vs. day 0. Estradiol: time × loading *p* = 0.5807, time *p* = 0.0221, loading *p* = 0.2344. Creatine‐Kinase M (CKM): no significant effect. NL: normal loading, HLS: hindlimb suspension. Dashed denote when measurements were not obtained.

### 3.4 Gene expression in the soleus muscle does not indicate sex‐based differences

**FIGURE 5:**
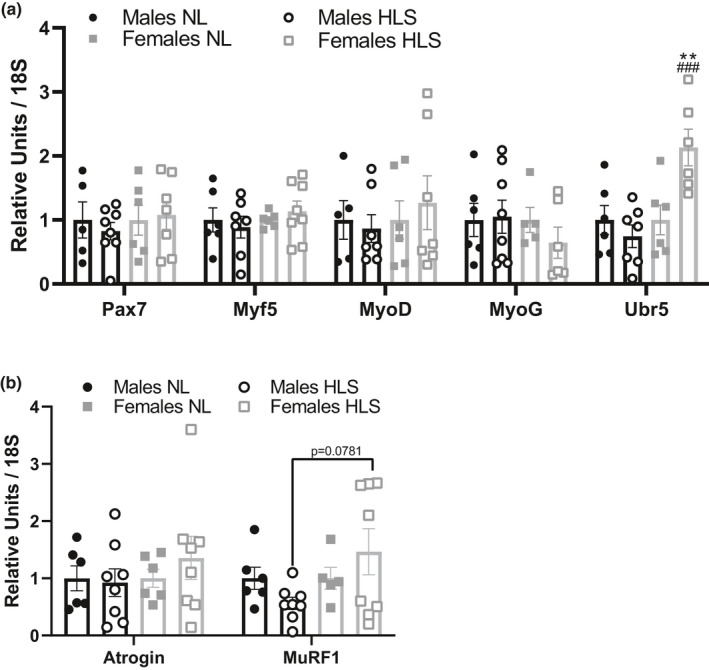
RTqPCR analysis of the soleus muscle. RTqPCR was performed on the soleus muscle to detect gene expression of Pax7, Myf5, MyoD, MyoG, and Ubr5 (a), Atrogin, muRF‐1 (b). Results are presented as mean ± SEM normalized with 18S as housekeeping gene, with *n* = 5–8 per group, and analyzed with two‐way ANOVAs followed by Tukey's multiple comparisons tests. **: *p* < 0.01 vs. sex‐matched NL animals, ###: *p* < 0.001 vs. loading‐matched males. NL: normal loading, HLS: hindlimb suspension

RTqPCR was performed on the soleus muscle and expression was normalized using 18S (Figure 5). mRNA content of genes linked to muscle catabolism (Figure 5b) did not present significant variation across groups (Factor sex × loading: Atrogin: *p* = 0.459, muRF‐1: *p* = 0.119), although a nonsignificant increase in MuRF‐1 was observed in HLS females compared to HLS males (*p* = 0.0798). mRNA content of genes related to myogenesis were not significantly different between groups (Figure 5a) (Factor sex × loading: *Pax7*: *p* = 0.565, *Myf5*: *p* = 0.443, *MyoD*: *p* = 0.546, *MyoG*: *p* = 0.437). However, analysis of Ubr5, an E3 ubiquitin ligase involved in muscle recovery, was upregulated in female HLS animals (Ubr5, effect of sex *p* = 0.0065, effect of loading *p* = 0.0702, effect of sex × loading *p* = 0.0065).

## 4 DISCUSSION

We studied the effects of 14 days of HLS in adult male and female rats. Exposure to HLS elicited a significant decline in grip strength, muscle mass, and myofiber cross‐section area (CSA) in all animals; however, there were also clear differences in the responses between the two sexes with females better maintaining their muscle function during disuse than males.

Unsurprisingly in both sexes, HLS led to weight loss (Figure 1a) and a comparable reduction in limb girth (Figure 1c) over the course of the 14‐day experiment. As previously reported (Caillaud et al., 2020; Carmo et al., 2016; Mortreux et al., 2018, 2019a, 2019c), maximal rear paw grip force (Figure 2b), steadily increased in normally‐loaded (NL) animals while it decreased during exposure to HLS. Among the NL groups, our results did not highlight significant differences between males and females, which confirms the results obtained in previous studies using healthy 100‐day old rats (Carmo et al., 2016). However, during disuse, females experienced a smaller decline in grip force than their male counterparts, and a three‐way interaction (sex‐time‐unloading condition) was detected. Females also seemed to conserve their force production capacities during dorsiflexion, while males did not (Figure 2d). We have previously observed this sexual dimorphism during exposure to partial weight‐bearing (Mortreux et al., 2018, 2019a, 2019b, 2019c; Semple et al., 2020), and greater decline in neuromuscular transmission efficiency and muscle strength has been shown to affect males rats exposed to 2 weeks of HLS more than females (Deschenes & Leathrum, 2016). However, our findings, both here and in the partial weight‐bearing model, are partially discordant to what has been reported in humans, where females tend to experience greater alterations in neuromuscular function than males (Deschenes et al., 2009, 2012; Yasuda et al., 2005) but men display increased fatigability (Clark et al., 2005; Deschenes et al., 2009; Fulco et al., 1999; Russ & Kent‐Braun, 2003). Yet, it is possible that the variable outcomes reported are due to discrepancies in unloading model, method, and time of the analysis. In particular, clinical bed rest studies that simulate weightlessness in humans, are often based on a single‐sex analysis involving men (Marusic et al., 2021). Based on the previous body of literature, our results could also be linked to substrate usage during force production, which was not explored in our study; since it has been shown that males rely more heavily on glycolytic ATP synthesis than females, which could contribute to the inability to sustain muscle force during submaximal challenges or fatigue protocols (Deschenes et al., 2009). Such mechanistic differences could explain the sex‐based discrepancies in force production such as those observed in our study.

Earlier studies performed in humans (Yasuda et al., 2005) and rats (Deschenes et al., 2018; Il’ina‐Kakueva, 2002; Yoshihara et al., 2019) have highlighted that disuse leads to similar muscle atrophy in males and females and that the soleus, a weight‐bearing muscle, is highly sensitive to mechanical unloading (Fitts et al., 2000, 2001; Hurst & Fitts, 2003; Thomason & Booth, 1990). Our current study revealed similar atrophy between sexes in both the gastrocnemius and TA muscles. However, when compared to controls, HLS males showed greater atrophy than female animals in both the soleus and EDL muscles (Figure 3a and Table 1), which could explain why females outperformed their counterparts during force testing (Figure 2c,d); especially since the TA is most powerful dorsiflexor of the ankle and largely responsible for force production. These data corroborate our findings of a greater decrement in muscle force and function in males than in females during disuse that was previously reported during exposure to partial gravity (Mortreux et al., 2018, 2019a, 2019b, 2019c; Semple et al., 2020). While consistent, our results may appear surprising since earlier investigations reported either no differences in the soleus structure after unloading (Il’ina‐Kakueva, 2002) or a greater loss in female rats compared to males after 7 days of unloading (Yoshihara et al., 2019). Therefore, we cannot exclude the possibility that muscle deconditioning does not follow a linear evolution, and that early changes might be associated with different underlying mechanisms that were not addressed with our current protocol.

Interestingly, HLS led to overall atrophy in the soleus both males and females regardless of myofiber type (Figure 3c,d). While this may appear surprising, based on previous literature (Ohira et al., 2002), this could potentially be due to the addition of using triple‐staining to better characterize hybrid myofibers. On the other hand, in the gastrocnemius muscle, we observed that HLS resulted in preferential atrophy in type 2 and hybrid myofibers in females, as opposed to preferential atrophy in type 1 myofibers in males. This disparity between sexes may be explained by an increased proportion of hybrid myofibers in the muscle of HLS males (Figure 3b) that was not observed in females. Moreover, these differences in fiber type might be responsible for the phenotypical divergence observed during force generation. Indeed, while females remained largely unaffected regardless of the loading/unloading status, HLS males failed to increase their force production over time (+0.43%) compared to their controls (+9.13%).

Recent clinical and preclinical studies have shown perturbations in satellite cell activity and abundance that accompany disuse‐induced muscle atrophy (McKenna & Fry, 2017) and implied the existence of sex‐based interaction in satellite cells abundance, proliferation, and differentiation (Lee et al., 2011; Neal et al., 2012; Song et al., 2013). Historical studies indicate that disuse atrophy is associated with myonuclear apoptosis (Allen et al., 1997) that would necessitate satellite cell fusion to restore. However, hindlimb suspension protocols have often shown decrements in satellite cell content concomitant with muscle atrophy (Babcock et al., 2015; Nakanishi et al., 2016); while clinical studies have yielded conflicting results, with studies showing both loss of satellite cell content with disuse (Arentson‐Lantz et al., 2016) and increased satellite cell content with immobilization (Suetta et al., 2013). Our data show a nonsignificant elevation in satellite cell content with hindlimb suspension in the soleus in both males and females, in addition to greater myonuclear content in hindlimb suspended animals. We note that female animals show a greater elevation in myonuclear density with suspension as compared to males (+34.2%, *p* = 0.052 in males, +50.1%, *p* = 0.0052 in females). The heightened myonuclear content during unloading in females may offer protection against disuse and contribute to the greater preservation of muscle function that we observed in the current study.

Sex‐hormones concentrations are strongly associated with muscle function. In males, testosterone promotes muscle mass and strength (Haizlip et al., 2015; Rosa‐Caldwell & Greene, 2019; Stein, 2013), and decrease in testosterone concentrations are associated with reduced muscle size (McHale et al., 2012; Nnodim, 2001; Sinha et al., 2014) and often observed during hindlimb unloading (Lin et al., 2016). Moreover, it has been shown that low‐dose testosterone supplementation can successfully enhance the impact of mitigating countermeasures in subjects exposed to long‐term bed rest (Dillon et al., 2018). Therefore, it is not surprising to detect a significant decrease in testosterone in HLS males at 14d compared to their baseline values (Table 2). In females, while testosterone levels could not be analyzed, we assessed the concentration of 17β‐estradiol, the most biologically active form, known to preserve muscle mass and regeneration (Greising et al., 2009; Larson et al., 2020), and for which deficiencies are linked to decreased muscle mass (Karvinen et al., 2021; Sitnick et al., 2006), strength (Kamanga‐Sollo et al., 2017), and quality (Qaisar et al., 2013). Indeed, muscle‐related outcomes were less impacted in female rats consistent with relative stability of estradiol concentration over time, but were not significantly correlated to their estrus cycle (*data not shown*, *p* = 0.727). However, it is important to note that all animals displayed greater variability in their hormonal concentration since our female rats were not cycled prior to baseline. This limitation should thus be addressed in future studies, as previous work highlighted that acyclic female mice exhibit significantly greater atrophy of the skeletal muscle than cycling mice during cancer cachexia progression (Hetzler et al., 2017) and that 17β‐estradiol could improve mitochondrial function and prevent cachexia's disruption (Counts et al., 2019).

Using RTqPCR, we were unable to detect differences between sexes or loading in the soleus muscle, the most affected of the hindlimb muscles in response to weightlessness and microgravity analogs (Fitts et al., 2000, 2001; Figure 5). While this may seem surprising due to the strong functional deficits elicited by our 14‐day HLS protocol, previous studies highlighted that gene expression is mostly modulated during the early stages of unloading (Mochalova et al., 2019) and often fails to detect significant differences after 14 days of exposure (Atherton et al., 2016). Therefore, it is possible that early changes were simply “missed”. Interestingly, prior works in mice have found females appear to have greater inductions of genes related to protein catabolism compared to males, and many of these differences appear to “normalize” after 7 days (Rosa‐Caldwell et al., 2021). Additionally, we cannot exclude that changes may have been present in other muscles such as the gastrocnemius and EDL. However, that same study found female mice appear to have greater overall basal protein synthetic rates throughout the early phases of disuse compared to males, which may help facilitate greater grip force maintenance throughout longer durations of disuse. On the contrary, we noted that Ubr5 expression, an E3 ubiquitin ligase involved in muscle recovery (Dablainville & Sanchez, 2019; Hughes et al., 2021), was significantly greater in HLS females compared to both NL animals and male counterparts. This unexpected increase could explain why females maintained better functional strength than males, suggesting a repair‐mechanism might already be activated in response to disuse.

In summary, our work highlights complex sex‐based differences in the muscles of rats exposed to simulated microgravity using hindlimb suspension. While all animals experienced muscle disuse in response to unloading, females were able to better maintain their muscle function compared to the males. Through these experiments, we hope to emphasize the need for researchers to assess the impact of reduced weight‐bearing in both sexes independently. This will allow us to better characterize the underlying mechanisms and therefore provide suitable, sex‐specific countermeasures for astronauts.

## CONFLICTS OF INTEREST

No conflict of interest, financial, or otherwise, are declared by the authors.

## AUTHOR CONTRIBUTIONS

MM, MRC, DS, IDS, and NTT performed the experiments, MM, MRC, DS, IDS, NTT, and CSF analyzed the results, MM and CSF prepared the figures, MM drafted the manuscript, MRC, NTT, CSF, and SBR edited the manuscript, MM, MRC, and SBR designed the research and experiments. All authors were involved in the revision of the manuscript and approved its final version.
